# Summer days: research culture and the neuroscience of taking a break

**DOI:** 10.1093/braincomms/fcae266

**Published:** 2024-09-02

**Authors:** Tara L Spires-Jones

**Affiliations:** Edinburgh, UK

## Abstract

Our editor discusses taking a vacation without a computer and some neuroscience evidence supporting the need for work–life balance.

## Graphical Abstract

**Graphical Abstract:**
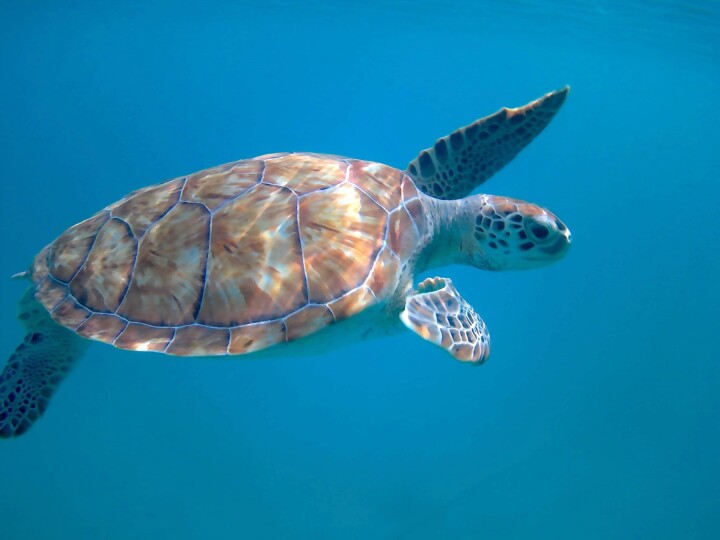

Welcome to volume 6, issue 5 of *Brain Communications*. I hope you have all enjoyed the summer. I’m writing this after an amazing two-week vacation in Barbados with my family and *without* my laptop. Many thanks to our Associate Editor Paul Skehel and our editorial team for keeping *Brain Communications* running during my break. Taking time to completely disconnect from work is not typical for some people in academia where we wear many hats and make commitments. However, research culture has started to shift with more open conversations about managing work load. In a survey by *Nature*, 75% of 1748 academics interviewed said that they have dialled back their work efforts over the past few years.^1^

There is strong evidence that adequate sleep and exercise boost cognition and that conversely stress impairs cognitive function, arguing for managing work–life balance and taking breaks.^2-4^ Recent data from Mueller *et al*.^5^ published in *Brain Communication* supports the link between sleep deficits and cognitive decline during aging. Spending two weeks snorkelling, scuba diving, taking walks in the jungle and not setting alarms to wake up early was certainly refreshing for me. As an added bonus, I managed to drown my smartphone in the Caribbean taking away my crutch to keep checking emails. This also helped with sleep as neuroscience research, including a recent study by Höhn *et al*.^6^ in *Brain Communications*, indicates that smartphone use before bedtime can impair sleep.

Alongside sleep and exercise, spending hours observing sea creatures (see ‘Graphical Abstract’ for a new friend from the trip) and experiencing another culture were inspiring. Despite the full inbox and to-do lists waiting at the end of vacation, being completely disconnected from work has left me feeling more creative and able to come back into projects with new ideas and renewed enthusiasm. I hope all of you dear readers also find time to take a break and disconnect.

The cover image for this issue comes from Schaper *et al*.^7^ and shows two brain networks derived from lesions causing parkinsonism versus seizures on opposing ends of a seesaw, illustrating an inverse relationship between these two common brain diseases.

## Competing interests

The authors report no competing interests.
